# Ultraviolet A1‐LED Phototherapy for the Treatment of Palmoplantar Pustulosis

**DOI:** 10.1111/phpp.13017

**Published:** 2025-02-13

**Authors:** Oki Watanabe, Yuki Enomoto, Mai Sakurai, Takashi Sakaida, Aya Yamamoto, Akimichi Morita

**Affiliations:** ^1^ Department of Geriatric and Environmental Dermatology Nagoya City University Graduate School of Medical Sciences Nagoya Japan

**Keywords:** palmoplantar pustulosis phototherapy, UVA1, UVA1 light‐emitting diodes

Pustulosis palmoplantaris (PPP) is a chronic inflammatory disease characterized by multiple new and old sterile pustules on the palms and soles. Topical corticosteroids and vitamin D3 are most frequently used to treat PPP. Phototherapy (e.g., PUVA, narrow‐band UVB, excimer light, and UVA1) and systemic therapy (cyclosporine, apremilast, guselkumab, etc.) are also used.

UVA1 emits wavelengths between 340 and 400 nm and penetrates the epidermis to reach the middle and deep dermal components. UVA1 phototherapy is highly effective in treating PPP [1, 2], atopic dermatitis, cutaneous T‐cell lymphoma, and dyshidrotic eczema, all of which involve pathogenetically relevant T‐cell infiltration. The present study investigated the effectiveness of UVA1 light‐emitting diode (UVA1‐LED) phototherapy for treating PPP and identifying the characteristics of effective cases. UVA1 phototherapy was applied to 16 patients with PPP. We developed a UVA1‐LED that theoretically consumes less electricity and produces much less heat, and demonstrated phototherapeutic efficacy for dyshidrotic palmoplantar eczema at a UVA1 dose of 30–60 J/cm^2^ once per week [3].

The study included 16 participants (Table 1) treated with UVA1‐LED for PPP between 2019 and 2023 in the Dermatology Clinic at Nagoya City University Hospital. The mean patient age was 58.8 ± 13.3 years (range: 26–85) and the male‐to‐female ratio was 1:15. This study was a prospective interventional study approved by the institutional review board of Nagoya City University Graduate School of Medical Sciences (approval number: 46‐18‐0014). After obtaining written informed consent from each participant, we administered phototherapy with the UVA1‐LED (Therabeam UVA1, Ushio, Japan). The initial dose was 30 J/cm^2^ once per week and the dose was increased in 30 J/cm^2^ increments up to a maximum of 90 J/cm^2^. Data collection included age, sex, disease duration, joint symptoms, smoking history, number of sessions, and maximum irradiation dose. Palmoplantar Pustulosis Area Severity Index (PPPASI) scores were evaluated at onset (pre‐PPPASI), and after five sessions, 10 sessions, and the final session (final‐PPPASI).

**TABLE 1:** Clinical characteristics of 16 patients with PPP.

	*n* = 16
Age, year (range)	58.8 (26–85)
Sex, *n*	Female 15, Male 1
Disease duration: year, mean (SD, range)	9.6 (9.7, 0.5–39)
Localization: palms and soles/soles, *n* (%)	13 (81.3%)/3 (18.8%)
Smoking history, *n* (%)	Current, 6 (37.5%), Former, 5 (31.3%), Non, 5 (31.3%)
Arthritis, *n* (%)	6 (37.5%)
Sessions, *n* (SD, range)	38.8 (22.8, 8–93)
Pre‐PPPASI, mean (SD, range)	13.3 (11.2, 6–54)
Five sessions‐PPPASI, mean (SD, range)	6.4 (8.4, 0.6–36)
Ten sessions‐PPPASI, mean (SD, range)	5.6 (6.4, 0.6–26)
Final‐PPPASI, mean (SD, range)	3.0 (5.4, 0–21.6)
Treatment response	
PPPASI 100, *n* (%)	4 (25%)
PPPASI 90, *n* (%)	10 (62.5%)
PPPASI 75, *n* (%)	12 (75%)

Abbreviation: PPPASI, Palmoplantar Pustular Psoriasis Area and Severity Index.

Statistical analysis was performed using Prism 10. We analyzed the PPPASI score, PPPASI responder rate, disease duration, smoking history, joint symptoms, and number of irradiations. Among the 16 patients, 6 were current smokers, 5 were nonsmokers, and 5 were former smokers. PPP was only on the soles of 3 patients and on both the palms and soles in 13 patients. The mean (SD) disease duration was 9.6 (9.7) years (range: 0.5–39 years) and the mean number of sessions was 38.8 (22.8, range: 8–93), with a maximum dose of 64.6 (22.3) J/cm^2^ for the palms and 75 (18.4) J/cm^2^ for the soles. The mean duration of time that patients received only topical treatment or no treatment after the final phototherapy session was 13.5 (12.8) months (range: 0–38 months). UVA1 phototherapy produced a significant improvement in the PPPASI score, with a mean score of 13.3 (11.2) before UVA1‐LED, 6.4 (8.4) at five sessions, 5.6 (6.4) at 10 sessions, and 3.0 (5.4) after the final session (Figure 1). The PPPASI score was significantly lower at five sessions (*p* = 0.045), 10 sessions (*p* = 0.0048), and after the final session (*p* < 0.0001).

**FIGURE 1:**
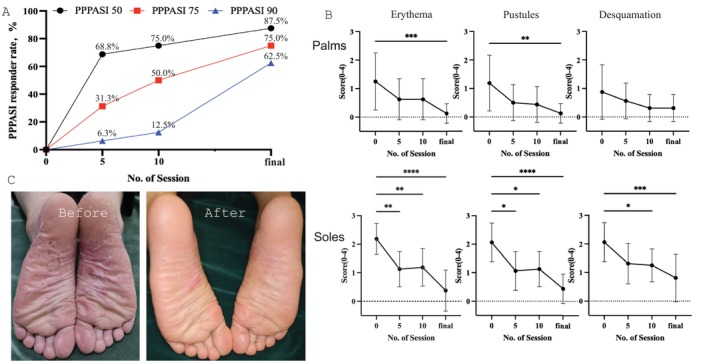
(A) PPPASI 50/75/90:50%, 75%, and 90%, reduction in Palmoplantar Pustular Psoriasis Area and Severity Index. (B) PPSI for erythema, pustules, and desquamation of the palms (top panel) and soles (bottom panel). (C) Representative case before and after UVA1‐LED phototherapy (×50, 90 J/cm^2^). *n* = 16, analyzed by Friedman's test. Additional statistics by Dunn's test. *P < 0.05, **P < 0.01, ***P < 0.0005, ***P < 0.0001.

As shown in the figure, a 75% reduction in the PPPASI score (PPPASI 75) was observed in 5 (31.3%) patients at five sessions, 8 (50.0%) patients at 10 sessions, and 12 (75.0%) patients after the final session. A 90% reduction in the PPPASI score (PPPASI 90) was observed in 1 (6.3%) patient at five sessions, 2 (12.5%) patients at 10 sessions, and 10 (62.5%) patients after the final session. A 100% reduction in the PPPASI score (PPPASI 100) was observed in 4 patients after the final session. Erythema, pustules, and desquamation of the palms and soles were evaluated using the Palmoplantar Severity Index (PPSI). The PPSI score of erythema, pustules of palms, and desquamation of the soles was lower after the final session, but the difference in desquamation of the palms after the final session was not significant. The PPPASI scores of current smokers, former smokers, and nonsmokers were all lower after the final session. The male‐to‐female ratio for pustulosis palmaris et plantaris is generally about 1:2, but in this study, the male‐to‐female ratio was significantly skewed at 1:15. Therefore, caution is required when interpreting the treatment effects for male patients. In the male cases in this study, the *Z*‐score of the PPPASI improvement rate was less than 0.5 at five, 10, and the final irradiation, and it is thought that the same treatment effects as the overall group were observed. Further accumulation and examination of cases are required to determine whether similar effects can be obtained in other male cases.

PPP treatment is challenging. In Japan, the anti‐interleukin 23p19 antibodies guselkumab and risankizumab were recently approved for use in PPP. Compared with the previously reported efficacy of guselkumab [4], UVA1‐LED phototherapy had a much faster onset of efficacy with 68.8% achieving PPPASI 50 and 31.3% achieving PPPASI 75 at five sessions. Acute side effects of UVA1 include erythema, tanning, polymorphic light eruption, itching, and recurrent herpes simplex infection, while chronic side effects include photoaging and skin cancer [5]. UVA1 phototherapy causes UV‐induced burning less frequently than UVB or PUVA therapy [6], and erythema is less likely to occur with appropriate emission. UVA1 phototherapy has fewer side effects and is safer than other phototherapies, and studies comparing UVA1 with narrowband UVB indicate that UVA1 is more effective [7].

In conclusion, UVA1 phototherapy is considered to be extremely effective for treating PPP and has mild side effects. Focusing on PPSI, however, no significant difference in palmar desquamation was observed after the final session, suggesting that UVA1‐LED phototherapy is highly effective for erythema and pustulosis of the palms and soles but less effective for desquamation of the palms.

## Conflicts of Interest

AM is the inventor and received a research grant from USHIO Inc. OW, YE, MS, TS, and AY have no conflict of interest.

## Data Availability

The data that support the findings of this study are available on request from the corresponding author. The data are not publicly available due to privacy or ethical restrictions.
